# Longitudinal associations of movement behaviours with body composition and physical fitness from 4 to 9 years of age: structural equation and mediation analysis with compositional data

**DOI:** 10.1186/s12966-023-01417-1

**Published:** 2023-02-07

**Authors:** Jairo H. Migueles, Christine Delisle Nyström, Dorothea Dumuid, Marja H. Leppänen, Pontus Henriksson, Marie Löf

**Affiliations:** 1 Department of Biosciences and Nutrition, Karolinska Institutet, 141 52 Huddinge, Sweden; 2 PROFITH “PROmoting FITness and Health through physical activity” research group, Department of Physical Education and Sports, Faculty of Sport Sciences, University of Granada, Granada, Spain; 3 Alliance for Research in Exercise, Nutrition and Activity, Allied Health & Human Performance, University of South Australia, Adelaide, SA Australia; 4 University of South Australia, Adelaide, SA Australia; 5 Faculty of Sport and Health Sciences, University of Jyväskylä, Jyväskylä, Finland; 6 Folkhälsan Research Center, Helsinki, Finland; 7 Department of Health, Medicine and Caring Sciences, Linköping University, 581 83 Linköping, Sweden

## Abstract

**Background:**

The associations of movement behaviours (physical activity [PA], sedentary behaviour [SB], and sleep) with body composition and physical fitness from pre-school to childhood, as well as the direction of the associations, could provide important information for healthy lifestyle promotion in children. This study investigated the longitudinal and bidirectional associations of movement behaviours with body composition and physical fitness measured at 4 and 9 years of age.

**Methods:**

This longitudinal study included baseline (*n* = 315, 4.5 [SD = 0.1] years) and follow-up data (*n* = 231, 9.6 [SD = 0.1] years) from the MINISTOP study. Movement behaviours were measured for 7 days using wrist-worn accelerometers, body composition with air-displacement plethysmography, and physical fitness with the ALPHA health-related fitness test battery. Cross-lagged panel models and mediation analyses were performed in combination with compositional data analysis.

**Results:**

We did not observe direct associations of the movement behaviours at 4 years with either body composition or physical fitness at 9 years (all *P* > 0.05). However, fat mass index at 4 years was negatively associated with vigorous PA (VPA), relative to remaining behaviours (VPA, β = − 0.22, *P* = 0.002) and light PA (LPA), relative to SB and sleep (β = − 0.19, *P* = 0.016) at 9 years. VPA (relative to remaining), moderate PA (MPA) (relative to LPA, SB, and sleep), and SB (relative to sleep) tracked from 4 to 9 years (all β ≥ 0.17, all *P* < 0.002), and these behaviours shared variance with fat mass index (all|β| ≥ 0.19, all *P* < 0.019), and aerobic, motor, and muscular fitness (all|β| ≥ 0.19, all *P* < 0.014) at 9 years. Mediation analysis suggested that the tracking of VPA (relative to remaining behaviours) from 4 to 9 years was negatively associated with fat mass index (β ≥ − 0.45, *P* = 0.012), and positively with aerobic fitness at 9 years (β ≥ 1.64, *P* = 0.016).

**Conclusion:**

PA and SB tracked from the pre-school years into childhood. Fat mass index at 4 years of age was negatively associated with VPA (relative to remaining behaviours) and LPA (relative to SB and sleep) at 9 years of age. The tracking of VPA was associated with lower fat mass index and higher aerobic fitness at 9 years of age. These findings suggest that higher levels of VPA in pre-school age, if maintained throughout childhood, may support the development of healthy body composition and aerobic fitness levels in later childhood.

**Supplementary Information:**

The online version contains supplementary material available at 10.1186/s12966-023-01417-1.

## Introduction

Daily time is spent across the movement behaviours of physical activity (PA), sedentary behaviour (SB), and sleep. Current evidence indicate that more time spent in PA is related to improved cognitive, mental, and physical health outcomes [1], while more time spent in SB and less time spent sleeping are associated with adverse mental and physical health outcomes in children and youth [2, 3]. However, to date, most studies have focused on a single movement behaviour (i.e., physical activity (PA), sedentary behaviour (SB), or sleep) without considering the interplay with the others (i.e., more daily time in one behaviour directly reduces the time in at least one of the other behaviours). There is also a lack of studies on how movement behaviours in the pre-school years (i.e., 3–5 years of age [1]) track into later childhood, and how they interact in their relationship with health outcomes such as body composition and physical fitness, which are known to be important indicators for children’s current and future health [2, 3]. A recent systematic review by Rollo et al. [4] investigating the composition of movement behaviours over 24-hours concluded that there is a need for high-quality longitudinal studies utilizing accelerometer assessed movement behaviours with a range of health behaviours across the lifespan. Interestingly, using compositional data analysis, which lowers the risk of multi-collinearity and considers the interplay between time spent in different behaviours, we have previously observed that more time spent in VPA, and less time spent in SB, light PA (LPA), and moderate PA (MPA) at 4 years of age was associated with benefits for body composition and physical fitness at 5 years of age [5]. However, it is unknown whether such associations are sustained later in childhood.

Accelerometers are commonly used to assess movement behaviours across the lifespan, and they are valid and feasible in pre-school and school-aged children [6, 7]. Few studies have examined longitudinal associations between accelerometer-assessed PA in pre-school aged children with body composition and physical fitness. In pre-schoolers, only three longitudinal studies are available with two of them reporting no association between PA and adiposity [6, 8]; whereas the third study found a positive association between moderate-to-vigorous PA (MVPA) and vigorous PA (VPA) with fat-free mass index [7]. With regards to physical fitness in pre-school children, positive longitudinal associations have been found between VPA and cardiorespiratory fitness [7, 8] as well as with muscular strength and motor fitness [7] in previous longitudinal studies. In school-aged children, longitudinal studies have found inverse associations between MVPA and adiposity [9, 10]; whereas another study reported a negative association between MVPA and fat-free mass index [11]. With regards to physical fitness in school-aged children a longitudinal study by Reisberg et al. [11] found positive associations between VPA at 6.6 years of age and cardiorespiratory fitness as well as upper- and lower-body muscular strength 1 year later. Thus, this demonstrates that more longitudinal studies assessing the interplay between movement behaviours with body composition and physical fitness are warranted spanning from pre-school to school-age children.

Although longitudinal associations between accelerometer-assessed PA and body composition in childhood have been reported, more studies are needed to reach conclusive statements on the directionality of the association. Cross-lagged panel models have the potential to elucidate the direction of such associations [12, 13]. Therefore, the aim of this study is to assess the bidirectional associations of movement behaviours with body composition and physical fitness from pre-school (4 years) to childhood (9 years) utilizing cross-lagged panel models. To the best of our knowledge, this is the first study investigating the longitudinal associations between accelerometer-assessed PA with body composition and physical fitness spanning from pre-school to school-aged children, and the first study combining cross-lagged panel models with compositional data analyses. We hypothesize a bidirectional association between the movement behaviours and body composition and physical fitness throughout childhood.

## Methods

### Study design and participants

This longitudinal cohort study used baseline (January to September 2014) and follow-up (February to September 2019) data from the MINISTOP trial (a population-based randomized controlled trial to promote PA and diet among 315 Swedish pre-schoolers) [14, 15]. In brief, MINISTOP was a two-arm, randomized controlled parallel population-based trial. Using Statistics Sweden’s population register, invitation letters for the MINISTOP trial were sent out to a population-based sample including parents of all 4-year-old children born between July 2009 and February 2010, residing in the county of Östergötland, Sweden. The 6-month mHealth intervention intended to help parents promote healthy eating and PA in their children [15]. The outcomes of the trial have been previously published with no effectiveness on the children’s PA levels [14]. Parents were included in the study if they had a healthy 4-year-old child, had the possibility to have their child measured at baseline (approximately at 4.5 years), and for at least one parent to able to speak and read Swedish sufficiently well to benefit from a mHealth app. In total, 315 children completed the baseline measures and were enrolled in the intervention study. Additionally, the children’s PA, body composition, and physical fitness were measured at a follow-up when the children were 9 years old. Out of the 231 children who accepted the invitation to the follow-up measurement, children without sufficient accelerometer data (*n* = 9), without complete body composition (*n* = 12) or physical fitness (*n* = 9) data were excluded from analyses, resulting in a final analytical sample of 201 children. Informed consent from parents were obtained. This study was approved by the Research Ethics Committee, Stockholm, Sweden (2013/1607–31/5;2013/2250–32;2018/220–32).

### Data collection

#### Movement behaviours

PA, SB, and sleep were continuously monitored with non-dominant wrist-worn accelerometers (ActiGraph GT3X+, Pensacola, FL, USA) for 7 days (24 h/day). Devices recorded accelerations at 50 Hz, and participants were instructed to only remove accelerometers for water-based activities. Children wearing the accelerometers for at least 16 hours during at least 1 day were included. Sensitivity analyses were conducted including only children who provided ≥3 valid days of accelerometer data (*n* = 190); as the results remained unchanged, the data including all participants were reported. The GGIR R package [16] was used to process the accelerometer data. In brief, the Euclidean Norm of the raw accelerations Minus One *G* was calculated over 5-s epochs; non-wear time periods were identified from the magnitude and variability of the raw accelerations measured at each accelerometer axis [17]; sleep and awake periods were identified using an automated algorithm [18, 19]; and awake time was subsequently classified into SB (< 35 m*g*), or PA of light (35–199 m*g*), moderate (200–699 m*g*), or vigorous (≥700 m*g*) intensity [20, 21].

#### Body composition

Body weight and height were measured using a standing stadiometer and a weight scale. Body mass index was calculated as kg/m^2^ and used to determine the weight status of the participants using the World Obesity Federation cut-points for age- and sex [22, 23]. Body composition was assessed using air-displacement plethysmography (www.cosmed.com) [24]. Body fatness was calculated from body volume applying appropriate densities for fat and fat-free mass [25]. Body composition outcomes included fat mass index (kg/m^2^) and fat-free mass index (kg/m^2^).

#### Physical fitness

Cardiorespiratory, motor, and muscular fitness were assessed with the PREFIT fitness test battery for pre-schoolers at the 4-year-old measurement [26], and the ALPHA fitness test battery at the 9-year-old timepoint [27]. The 20-m shuttle run test was used for cardiorespiratory fitness, the 4 × 10-m shuttle run test for motor fitness, the handgrip strength test for upper-body strength, and the standing long jump test for lower-body strength. The best of two attempts was recorded, except for the 20-m shuttle run test that was performed once [7, 28]. A relative measure of upper-body strength was obtained from dividing the kg squeezed in the handgrip test by body weight. As participants carry their own body weight in the performance for the standing long jump test, lower-body strength is originally relative to body weight. Then, a single measure of the muscular fitness was calculated as the average of the z-scores for the upper- and lower-body strength as relative indicators and used in the analyses.

### Statistics

Descriptive characteristics of the participants were reported as mean and standard deviation (SD) or frequencies as appropriate. We performed a structural equation model delineating concurrent relationships (zero-order correlations) between the movement behaviours and body composition outcomes, as well as cross-lagged effects of the movement behaviours at baseline on body composition at follow-up and vice versa. A similar model was performed for the physical fitness outcomes. This approach is widely used in the analysis of longitudinal data to test longitudinal predictive effects between variables while accounting for auto-regressive effects of past behaviour on future respective behaviour [29, 30]. Based on previous studies [5, 7, 28] in the same participants, sex, age (at baseline and follow-up), maternal education (university level or below) and group allocation (intervention or control) were included as covariates in the models. Sensitivity analyses were performed including energy intake at 4 and 9 years of age derived from a food-frequency questionnaire. The Satorra-Bentler scaled chi-square test statistic assessed goodness-of-fit of the model (*P* > 0.05), and approximate model fit was examined using the recommendations of Hu and Bentler [31], i.e., comparative fit index (CFI) ≥ 0.95; root mean square error of approximation (RMSEA) ≤ 0.06, standardized root mean square residual (SRMR) ≤ 0.08. Additionally, mediation analysis was used to investigate whether the tracking of the movement behaviours from 4 to 9 years old were associated with body composition and physical fitness. The movement behaviours at 4 years were included as explanatory variables, the movement behaviours at 9 years as mediator, and the body composition and physical fitness outcomes at 9 years as dependent variables. Although the mediator and outcome were both measured at the same time point, our causal model depicted the movement behaviours as preceding the body composition and physical fitness outcomes. Mediation models were also adjusted for sex and age (at baseline and follow up), maternal education, and group allocation. Standardized beta-coefficients (β) were examined for significance, magnitude, and direction of the relationships. Modelling was performed using the lavaan R package (version 0.6.11).

In line with a recent expert consensus on the analytical approaches for accelerometer-assessed movement behaviours [32], we followed the compositional data analysis standards [33, 34] in combination with structural equation modelling and mediation analysis. Compositional data analysis accounts for the relative nature of device-assessed movement behaviours by quantifying the effect of increasing a specific behaviour while reducing at least one of the others. As such, compositional descriptive statistics consisted of the geometric mean (normalized mean to the average day duration) and the variation matrix. The variation matrix summarizes the variability structure of the data by means of log-ratio variances, the lower the values the higher the inter-dependence between that pair of behaviours.

For the regression models, the movement behaviours were expressed as a set of isometric log-ratio (ILR) coordinates [33] as follows:

1 $${ilr}_1=\sqrt{\frac{4}{5}}\mathit{\ln}\frac{Vigorous}{{\left( Moderate\cdot Light\cdot Sedentary\cdot Sleep\right)}^{{}^{1}\!\left/ \!{}_{4}\right.}}$$

2 $${ilr}_2=\sqrt{\frac{3}{4}}\mathit{\ln}\frac{Moderate}{{\left( Light\cdot Sedentary\cdot Sleep\right)}^{{}^{1}\!\left/ \!{}_{3}\right.}}$$

3 $${ilr}_3=\sqrt{\frac{2}{3}}\mathit{\ln}\frac{Light}{{\left( Sedentary\cdot Sleep\right)}^{{}^{1}\!\left/ \!{}_{2}\right.}}$$

4 $${ilr}_4=\sqrt{\frac{1}{2}}\mathit{\ln}\frac{Sedentary}{Sleep}$$

The ILR coordinates represent the effect of increasing a behaviour while decreasing the rest of behaviours, accounting for the relative nature between behaviours and the time constrain of the 24-hour day, to allocate time across behaviours (i.e., at least one behaviour should decrease to increase another). For example, ilr_1_ represents the effect of increasing VPA while proportionally decreasing MPA, LPA, SB, and sleep time.

## Results

**Fig. 1:**
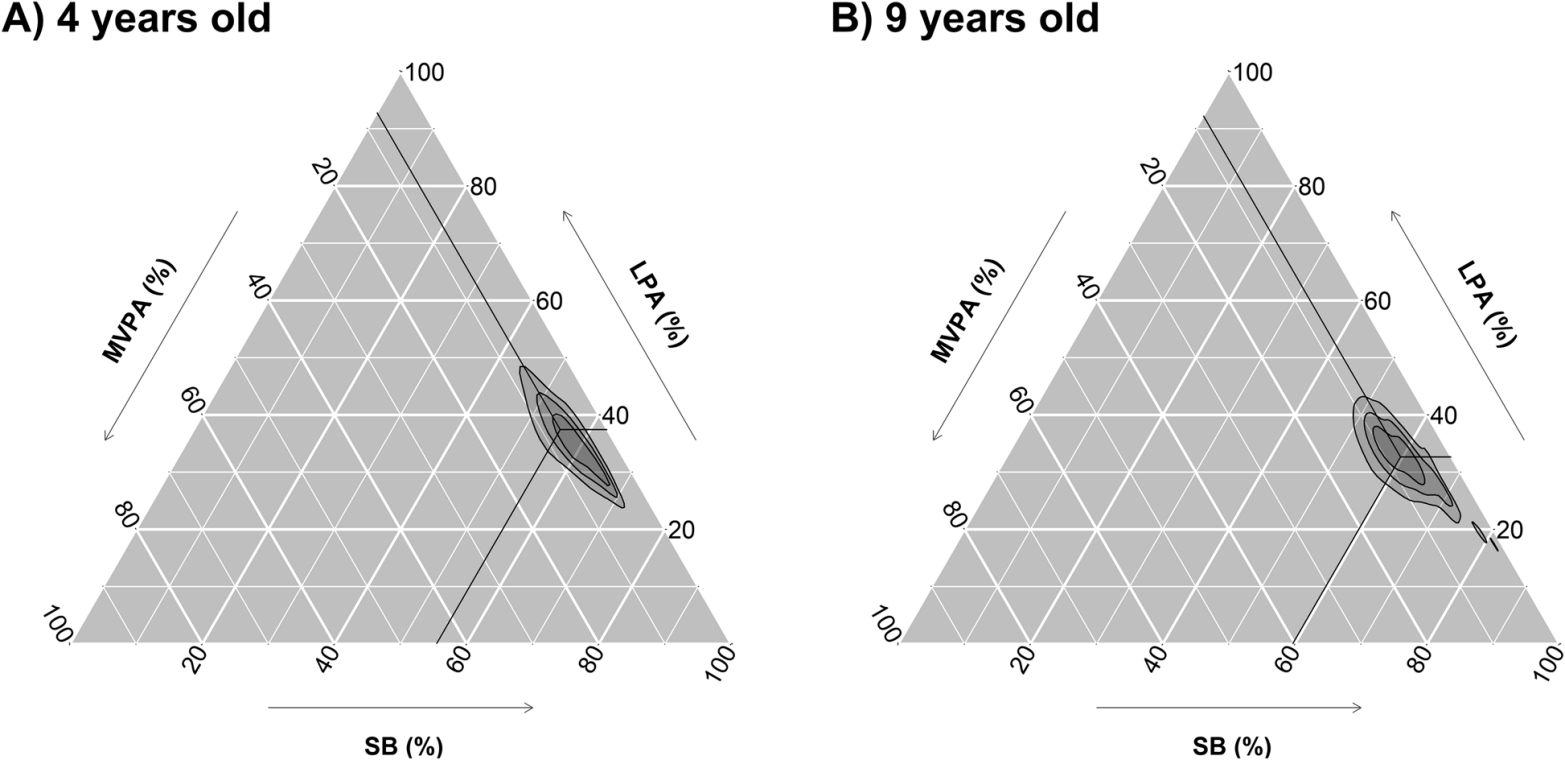
Ternary plots for the daily time-use in the movement behaviours at 4 years old (**A**), 9 years old (**B**) (*n* = 217). The crosshair marks represent the geometric mean of the awake time behaviours at (**A**) 4 years old (i.e., VPA: 10 min/day, MPA: 55 min/day, LPA: 346 min/day, SB: 512 min/day, Sleep: 518 min/day) and (**B**) 9 years old (i.e., VPA: 13 min/day, MPA: 54 min/day, LPA: 294 min/day, SB: 537 min/day, Sleep: 543 min/day). Concentric rings represent the 25, 50 and 75% confidence regions. MVPA: moderate-to-vigorous physical activity, VPA: Vigorous physical activity, MPA: Moderate physical activity, LPA: light physical activity, SB: sedentary behaviour

Descriptive sociodemographic, body composition, and physical fitness characteristics of participants are summarized in Table 1. The movement behaviour compositions at 4 years and 9 years are graphically presented in ternary plots in Fig. 1. The geometric mean of the movement behaviours investigated at 4 years old was 10 min of VPA, 55 min of MPA, 346 min of LPA, 512 min of SB and 518 min of sleep per day. Correspondingly, the geometric mean at 9 years old was 13 min of VPA, 54 min of MPA, 294 min of LPA, 537 min of SB, and 543 min of sleep per day. The covariance matrices for the movement behaviours at 4 and 9 years old are presented in Table S1 (Supplementary material). The children who were not included in this study (*n* = 114) showed similar baseline characteristics to those included in the analysis (*n* = 201) (Supplementary material, Table S2 and Fig. S1). Of the children included in the analysis, 96% (*n* = 193) provided valid accelerometer data for at least 3 days at 4 years and 99% (*n* = 198) at 9 years. Mean (SD) daily wear time was 22.4 (2.1) hours per day at 4 years and 23.6 (1.0) hours per day at 9 years.

**Table 1:** Characteristics of participating children at 4 and at 9 years of age

	4 years old		9 years old	
	n	Value	n	Value
Age (yrs)	315	4.5 (0.1)	231	9.6 (0.1)
Height (cm)	315	107.6 (4.2)	231	139.7 (6.3)
Weight (kg)	315	18.3 (2.5)	231	33.5 (7.1)
Weight status *n (%)*	315		231	
Underweight		27 (8.6)		22 (9.5)
Normal weight		260 (82.5)		176 (76.6)
Overweight		24 (7.6)		29 (12.6)
Obesity		4 (1.3)		4 (1.7)
Mother education level *n (%)*	315			
University or higher		223 (70.8)		
Below university		92 (29.2)		
*Body composition*				
FFMI (kg/m^2^)	303	11.6 (1.0)	230	13.5 (1.1)
FMI (kg/m^2^)	303	4.1 (0.9)	230	3.6 (1.9)
*Physical fitness*				
Aerobic (laps)	315	15.8 (1.4)	231	17.1 (2.5)
Speed-Agility (s)	303	11.6 (1.0)	230	13.5 (1.1)
Upper-body strength (kg)	303	26.0 (4.4)	230	20.0 (7.4)
Lower-body strength (cm)	303	4.1 (0.9)	230	3.6 (1.9)

Data presented as mean (SD) unless otherwise stated

### Cross-lagged panel models

**Fig. 2:**
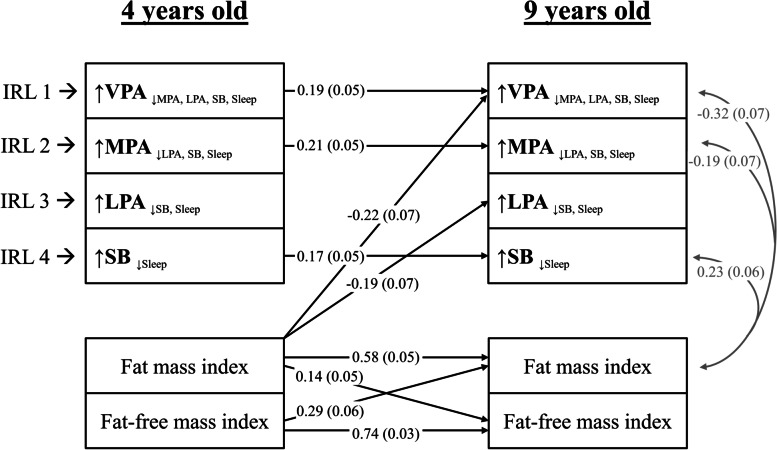
Lagged associations of the 24-h movement behaviour composition with body composition and concurrent relationships at 9 years old (*n* = 201). The model is adjusted for sex and age at baseline and follow up, group allocation, and maternal education. Gray lines indicate shared covariance. Only statistically significant paths (*P* < 0.05) are shown. Model fit indices: CFI: 0.99, RMSA: 0.05, SRMR: 0.025. Data presented are standardized coefficients (standard error). ILR: isometric log ratio, VPA: vigorous physical activity, MPA: moderate physical activity, LPA: light physical activity, SB: sedentary behaviour, CFI: comparative fit index, RMSA: root mean square error of approximation, SRMR: standardized root mean square residual

Figure 2 shows the cross-lagged panel model for the longitudinal associations between the movement behaviours and the body composition outcomes (i.e., fat mass index and fat-free mass index). This model reported a good fit (χ^2^ = 20.19, df = 12, *p* = 0.064; RMSEA = 0.058 [CI: 0.099–0.335]; CFI = 0.989). None of the movement behaviours at 4 years old were associated with body composition at 9 years old directly. However, higher fat mass index at 4 years old was associated with lower VPA relative to the remaining behaviours (β = − 0.22, *P* = 0.002) and lower LPA relative to SB and sleep (β = − 0.19, *P* = 0.016). The ILR_1_ (VPA relative to the remaining behaviours), ILR_2_ (MPA relative to LPA, SB, and sleep), and ILR_4_ (SB relative to sleep) at 4 years old were positively associated with the corresponding variables at 9 years old (β = 0.19, *P* < 0.001; β = 0.21, *P* < 0.001; and β = 0.17, *P* = 0.001, respectively). Likewise, the ILR_1_, ILR_2_, and ILR_4_ at 9 years old shared variance with fat mass index at the same timepoint (β = − 0.32, *P* < 0.001; β = − 0.19, *P* = 0.019; and β = 0.23, *P* = 0.001, respectively). Finally, the body composition outcomes at 4 years old were associated with the body composition outcomes at 9 years old (all β’s > 0.14, all *P*’s < 0.006).

**Fig. 3:**
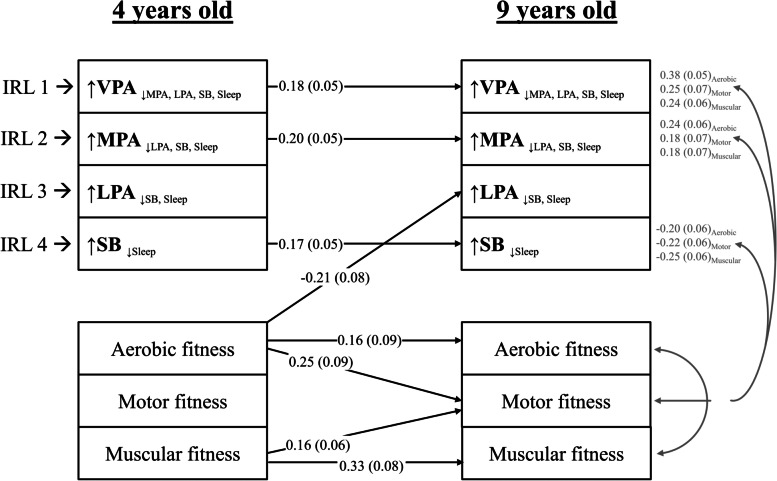
Lagged associations of the 24-h movement behaviour composition with physical fitness and concurrent relationships at 9 years old (*n* = 201). The model is adjusted for sex and age at baseline and follow up, group allocation, and maternal education. Gray lines indicate shared covariance. Only statistically significant paths (*P* < 0.05) are shown. Model fit indices: CFI: 0.99, RMSA: 0.03, SRMR: 0.02. Data presented are standardized coefficients (standard error). ILR: isometric log ratio, VPA: vigorous physical activity, MPA: moderate physical activity, LPA: light physical activity, SB: sedentary behaviour, CFI: comparative fit index, RMSA: root mean square error of approximation, SRMR: standardized root mean square residual

The longitudinal associations between the movement behaviours and the physical fitness outcomes (i.e., aerobic, motor, and muscular fitness) are shown in Fig. 3. This model reported a good fit (χ^2^ = 14.73, df = 12, *p* = 0.256; RMSEA = 0.034 [CI: 0.000–0.082]; CFI = 0.996). None of the movement behaviours at 4 years old were associated with physical fitness at 9 years old directly. However, higher aerobic fitness at 4 years old was associated with lower ILR_3_ (representing LPA relative to SB and sleep; β = − 0.22, *P* = 0.008). Furthermore, the ILR_1_, ILR_2_ and ILR_4_ at 4 years old were positively associated with the corresponding variables at 9 years old (β = 0.18, *P* = 0.001; β = 0.20, *P* < 0.001; and β = 0.17, *P* = 0.001, respectively). The ILR_1_, ILR_2_ and ILR_4_ coordinates at 9 years old shared variance with at least one physical fitness component at the same timepoint (all *P* < 0.014). Finally, aerobic, and muscular fitness at 4 years old were associated with aerobic, motor, and muscular fitness at 9 years old (all β > 0.16, all *P* < 0.048).

### Mediation analysis

**Fig. 4:**
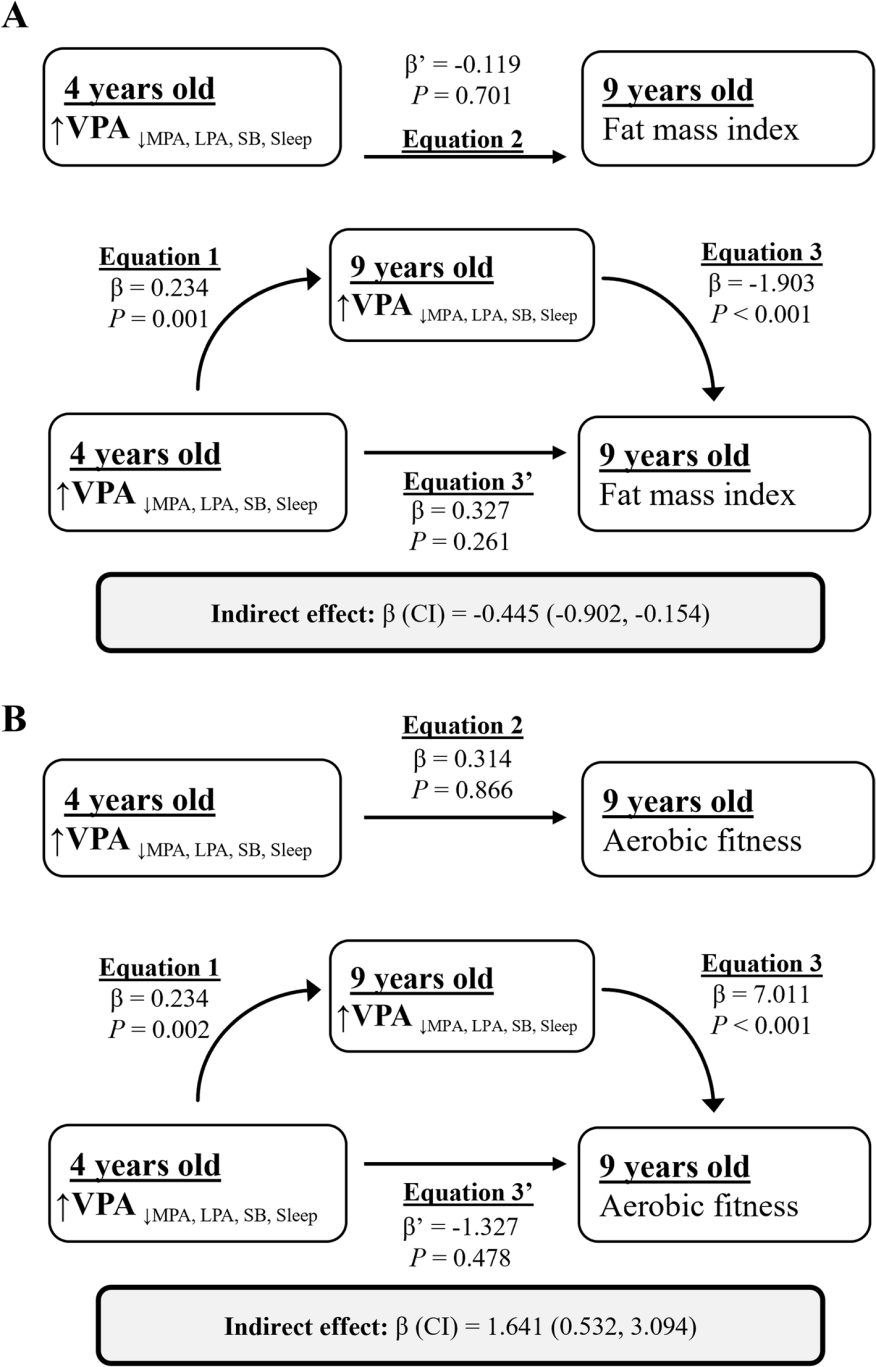
Simple mediation analysis of VPA at 4 years of age with fat mass index (**A**) and aerobic fitness (**B**) via VPA at 9 years of age (*n* = 201). The model is adjusted for sex and age at baseline and follow up, group allocation, maternal education, and the other isometric log ratios to account for the 24-hour time-use composition. Equation 1 * Eq. 2 shows the natural indirect effect pathway, and Eq. 3’ shows the natural direct effect pathway. VPA: vigorous physical activity, MPA: moderate physical activity, LPA: light physical activity, SB: sedentary behaviour

**Fig. 5:**
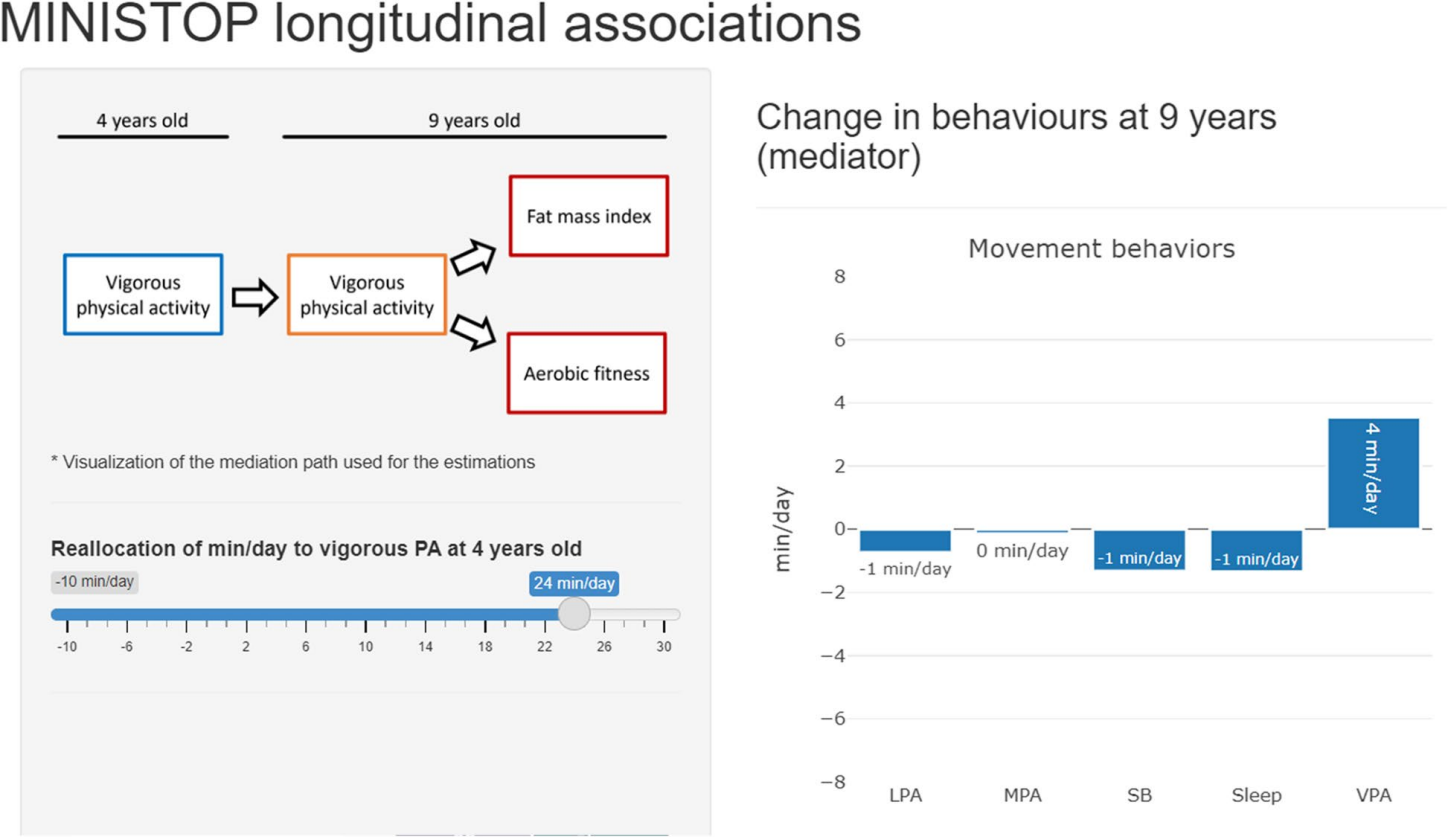
Estimated change in fat mass indez at 9 years of age upon reallocating daily time to VPA from MPA, LPA, SB, and sleep at 4 years of age. The visualization of the app can be visited at: https://jhmigueles.shinyapps.io/MINISTOP/. VPA: vigorous physical activity, MPA: moderate physical activity, LPA: light physical activity, SB: sedentary behaviour

Mediation analyses were conducted to investigate the indirect association of the ILR coordinates at 4 years old with body composition and physical fitness outcomes using the ILR coordinates at 9 years old as a mediator. We observed statistically significant mediation paths for the association of VPA relative to the remaining behaviours with fat mass index (β = − 0.45, *P* = 0.012) and aerobic fitness (β = − 1.64, *P* = 0.016) (Fig. 4). The change in fat mass index and aerobic fitness estimated from these models after manipulating VPA relative to the remaining behaviours at 4 years old can be visualized in a shiny app (https://jhmigueles.shinyapps.io/MINISTOP/, code available at https://github.com/jhmigueles/MINISTOP) (Fig. 5). The total and direct effect in these mediation analyses were not statistically significant. The rest of the ILRs (movement behaviours) were not associated with any of the outcomes in the mediation analyses performed.

### Sensitivity analysis

Results remained virtually the same after additional adjustment for average energy intake at 4 and 9 years of age derived from a food-frequency questionnaire (Supplementary material, Figs. S2 and S3).

## Discussion

In the present study, more VPA (relative to MPA, LPA, SB, and sleep) at 4-years of age was indirectly associated (i.e., the behaviour tracks from 4 to 9 years) with better body composition and physical fitness at 9-years of age. There was no evidence of LPA (relative to SB and sleep) being associated with body composition or physical fitness throughout childhood. Likewise, a lower fat mass index at 4 years was associated with higher VPA (relative to the remaining behaviours) and LPA (relative to SB and sleep) at 9 years of age. A shiny app [35] (https://jhmigueles.shinyapps.io/MINISTOP/) was built to visualize the estimated change in the outcomes at 9 years old as a result of manipulating VPA relative to the remaining behaviours (code available at https://github.com/jhmigueles/MINISTOP). The only purpose of this shiny app is for the communication/visualization of the model statistics presented in the mediation analysis as minutes per day, and how this change in the minutes per day is associated with fat mass index and aerobic fitness.

A recent systematic review concluded that there is a need for high-quality longitudinal studies investigating movement behaviours and health indices [4]. To the authors knowledge, this is the first study investigating the longitudinal associations between movement behaviours with body composition and physical fitness spanning from pre-school to school-aged children; as well as the first study using compositional data transformations (ILRs) in combination with structural equation modelling and mediation analyses. The longitudinal design, the referent measurement methods, and the novel analytical approach make this study unique.

In the present study we found that movement behaviours, body composition, and physical fitness track from pre-school to childhood. Our results are in line with previous studies who have found evidence of moderate tracking and moderate-to-large tracking of PA and SB, respectively from early childhood to middle childhood [36]. Furthermore, we observed direct associations between fat mass index and fat-free mass index as well as cardiorespiratory fitness and muscular strength between 4 and 9 years of age. To the best of our knowledge no studies have investigated the tracking of body composition and physical fitness from the pre-school years into childhood. With regards to body composition, a previous study in Swedish children found positive associations between fat and fat-free mass index measured using air displacement plethysmography at 12 weeks of age and at 4 years of age [37]. Furthermore, tracking of adiposity indicators (i.e., body mass index or skinfolds) have been observed in school-aged children [38–41]. Aerobic fitness has also been found to track in the pre-school years [42], in school aged-children [40, 41], and from childhood to adolescence [43], whereas muscular strength has previously been found to track in school-aged children [40, 41]. Thus, the positive longitudinal associations observed in the current study demonstrate the importance for health promotion interventions in the pre-school years to create and support healthy movement behaviours, body composition, and physical fitness levels in later childhood.

To date, the majority of longitudinal studies in pre-school and school-aged children have not considered the interplay between movement behaviour with body composition and physical fitness [6–11]. Using compositional analysis, we found indirect associations between more VPA relative to all the other behaviours at 4-years of age with better fat mass index and aerobic fitness at 9-years of age. These associations were fully mediated by the VPA performed at 9 years of age, which suggests that the promotion of VPA should start early (in pre-school age) and be maintained throughout childhood in order to impact later body composition and physical fitness in children. Previous cross-sectional studies in school-aged children using compositional data analysis have shown the importance of MVPA in relation to body composition and/or aerobic fitness [44–47]. Thus, these results further highlight the importance of the promotion of creating healthy movement behaviours in the pre-school years.

Furthermore, we found that a higher fat mass index at 4-years of age was directly associated with lower VPA relative to the other behaviours (ILR1), and LPA relative to SB and sleep (ILR3) at 9-years of age. In school-aged children contrasting results have been found regarding prospective bidirectional relationships between objectively measured PA and adiposity. In a study by Tanaka et al. [48] a negative association was observed between adiposity at 7-years of age with MVPA at 9-years of age, but not vice versa. Similar results were also observed in a study in 8- to 11-year-old children where a higher fat mass index at baseline was associated with a decrease in MVPA, but not the opposite [49]. Two other studies in school-aged children found no prospective associations between fat mass index and MVPA [9, 50]; however, Marques et al. [50] observed an inverse longitudinal relationship between MVPA and fat mass index. Our study is the first to use compositional data analyses to investigate the prospective bidirectional associations of objectively measured PA and body composition. Furthermore, the aforementioned studies have all utilized standard analytical approaches which could bias the findings due to multicollinearity [51, 52]. Thus, in order to lower the risk of multicollinearity, compositional data analysis has been suggested in order to accurately assess the reallocation of time across movement behaviours [33, 34]. Therefore, more prospective studies starting in the pre-school years utilizing compositional data analysis are needed to further elucidate the bidirectional relationships between objectively assessed PA and body composition. One limitation to compositional data analysis is the difficulty of interpreting ILRs in a meaningful way and we have demonstrated how this can be overcome by use of an interactive shiny app which enables the user to interpret the movement behaviours ILRs in minutes/day.

Interestingly, in the present study we found a direct association between fat mass index at 4-years of age with PA (ILR_1_) at 9-years of age; however, we did not find a direct association between PA at 4-years of age with fat mass index at 9-years of age. A possible reason for this is that fat mass is defined by a combination of genetic and environmental factors, thus it may be hypothesized that the genetic factors defining fat mass may also predispose children to perform less PA from the pre-school to school years. With regards to PA, we are only measuring an environmental factor, thus the association with later body composition may appear weaker as we may be missing part of the information. However, in this study we observed an indirect relationship between VPA relative to the other behaviours at 4-years of age with a lower fat mass index at 9-years of age. This suggests that the promotion of VPA should be effectively maintained throughout childhood, starting as early as the pre-school years. This is promising as PA is an environmental factor which is modifiable and future health promotion initiatives and interventions should focus on increasing VPA throughout childhood. In this respect, it is relevant to note our previously reported results from interviews with preschool teachers [53]. Our findings showed that the pre-school teachers perceived children’s VPA as low in their daily work and agreed that PA of higher intensities (e.g., climbing, running) could be offered more often. However, they expressed that environmental and structural barriers such as sub-optimal facilities or time constraints refrained them from doing so. Targeting such barriers could be important for future interventions to promote VPA in pre-school aged children.

### Strengths and limitations

Strengths of this study include: the longitudinal design, relatively long follow-up period (5 years) spanning from the pre-school years into mid childhood, accurate and reliable measures of body composition (i.e., air displacement plethysmography) [54] and physical fitness [27]. This study is further strengthened through the advanced processing techniques for the ActiGraph data [16, 32, 55] as well as the use of compositional data analysis in combination with structural equation models and mediation analyses [32]. This study is limited through the relatively small sample size as well as the fact that MINISTOP was originally a randomized controlled trial. However, it is important to highlight that the intervention took place between 4.5 to 5 years of age and that the intervention was multicomponent with the major focus on diet with no effect on PA or SB observed using accelerometry at either the 5- or 5.5-year follow-ups [14, 15]. Furthermore, the allocation group was used as confounder in the analyses of this study. There might be residual confounding not considered in the analyses, e.g., dietary information, however, adjusting for energy intake at 4 and 9 years collected using a food-frequency questionnaire did not change our estimates (Supplementary Material, Figs. S2 and S3). For the mediation analyses, our causal framework considered movement behaviours at 9 years to precede the body composition/physical fitness outcomes which were measured at the same time point. Thus, the mediation effects should be considered as cross-sectional associations. Noteworthy, we also had a large variation in movement behaviours as well as body composition and physical fitness variables which is essential when investigating associations. Nevertheless, the generalizability of the results may be limited by the fact that, although efforts were made to recruit a representative sample, participating parents had a slightly higher educational status than the general population [56]. A further limitation is that the algorithm used to estimate sleep in this study is validated only in adults, which might compromise the validity of the measure for children. However, we did not find any open-source algorithm to detect sleep from wrist-worn accelerometers in pre-school and school-aged children [57]. The slightly less time spent sleeping in 4 years vs 9 years should be interpreted as more occurrence of postural changes in preschoolers than in older children, whether this indicate actual lower sleep remains to be investigated. Similarly, we used cut-points for PA intensities validated in children at both time points, even though there are other alternatives specific for pre-schoolers [57]. It is important to note that the algorithms for wrist-worn accelerometer data in children have not been cross-validated, and the evidence for their use is compromised. Therefore, we decided to be consistent in the methods used at both time points and avoid any source of error originating from different algorithms. As a result, caution is advised when comparing the sleep and PA estimates (min/day) calculated at 4 years and 9 years of age. Although the absolute values for the estimates are not comparable across cut-points [58], the association with body composition and physical fitness in school-aged children [59] and pre-schoolers [60] is consistent across different cut-points.

## Conclusion

PA and SB were found to track from the pre-school years into childhood. Fat mass index at 4 years of age was negatively associated with VPA (relative to remaining behaviours) and LPA (relative to SB and sleep) at 9 years of age. Furthermore, the tracking of VPA was associated with lower fat mass index and higher aerobic fitness at 9-years of age. These findings suggest that higher levels of VPA in pre-school age, if maintained throughout childhood, may support the development of healthy body composition and aerobic fitness levels in later childhood.

## Supplementary Information

**Additional file 1: Table S1.** Covariance matrices for the daily time-use in movement behaviours. **Table S2.** Comparison of baseline demographic characteristics (4 years old) of children included and not included in this study. **Fig. S1.** Ternary plots for the daily time-use in the movement behaviours at 4 years old in the included children (A) and the dropouts (B). The crosshair marks represent the geometric mean of the awake time behaviours at 4 years old in the (A) included children (i.e., VPA: 10 min/day, MPA: 55 min/day, LPA: 348 min/day, SB: 512 min/day, Sleep: 515 min/day) and (B) 9 years old (i.e., VPA: 8.4 min/day, MPA: 51 min/day, LPA: 272 min/day, SB: 562 min/day, Sleep: 645 min/day). Concentric rings represent the 25, 50 and 75% confidence regions. The difference in the time-use composition of VPA, MPA, LPA, SB, and sleep is not significantly different between the included children and the dropouts (P = 0.628). **Fig. S2.** Lagged associations of the 24-h movement behaviour composition with body composition and concurrent relationships at 9 years old (n = 196). The model is adjusted for sex and age at baseline and follow up, group allocation, maternal education, and energy intake at baseline and follow up. Gray lines indicate shared covariance. Only statistically significant paths (*P* < 0.05) are shown. Model fit indices: CFI: 0.99, RMSA: 0.05, SRMR: 0.025. Data presented are standardized coefficients (standard error). **Fig. S3.** Lagged associations of the 24-h movement behaviour composition with physical fitness and concurrent relationships at 9 years old (n = 196). The model is adjusted for sex and age at baseline and follow up, group allocation, maternal education, and energy intake at baseline and follow up. Gray lines indicate shared covariance. Only statistically significant paths (P < 0.05) are shown. Model fit indices: CFI: 0.99, RMSA: 0.05, SRMR: 0.025. Data presented are standardized coefficients (standard error).

## Data Availability

The datasets generated and/or analyzed during the current study are not publicly available but are available from the corresponding author on reasonable request.

## Abbreviations

CFI: Comparative fit index
ILR: Isometric log-ratio
LPA: Light physical activity
MPA: Moderate physical activity
MVPA: Moderate-to-vigorous physical activity
PA: Physical activity
RMSEA: Root mean square error of approximation
SB: Sedentary behaviour
SD: Standard deviation
SRMR: Standardized root mean square residual
VPA: Vigorous physical activity
