# Supplementary material for: Longitudinal associations of movement behaviours with body composition and physical fitness from 4 to 9 years of age: structural equation and mediation analysis with compositional data
Source: Int J Behav Nutr Phys Act. 2023 Feb 7;20:11. doi: 10.1186/s12966-023-01417-1 (PMC9903529; doi:10.1186/s12966-023-01417-1)
Supplement: Supplementary file 1 — Additional file 1: Table S1. Covariance matrices for the daily time-use in movement behaviours. Table S2. Comparison of baseline demographic characteristics (4 years old) of children included and not included in this study. Fig. S1. Ternary plots for the daily time-use in the movement behaviours at 4 years old in the included children (A) and the dropouts (B). The crosshair marks represent the geometric mean of the awake time behaviours at 4 years old in the (A) included children (i.e., VPA: 10 min/day, MPA: 55 min/day, LPA: 348 min/day, SB: 512 min/day, Sleep: 515 min/day) and (B) 9 years old (i.e., VPA: 8.4 min/day, MPA: 51 min/day, LPA: 272 min/day, SB: 562 min/day, Sleep: 645 min/day). Concentric rings represent the 25, 50 and 75% confidence regions. The difference in the time-use composition of VPA, MPA, LPA, SB, and sleep is not significantly different between the included children and the dropouts (P = 0.628). Fig. S2. Lagged associations of the 24-h movement behaviour composition with body composition and concurrent relationships at 9 years old (n = 196). The model is adjusted for sex and age at baseline and follow up, group allocation, maternal education, and energy intake at baseline and follow up. Gray lines indicate shared covariance. Only statistically significant paths (P < 0.05) are shown. Model fit indices: CFI: 0.99, RMSA: 0.05, SRMR: 0.025. Data presented are standardized coefficients (standard error). Fig. S3. Lagged associations of the 24-h movement behaviour composition with physical fitness and concurrent relationships at 9 years old (n = 196). The model is adjusted for sex and age at baseline and follow up, group allocation, maternal education, and energy intake at baseline and follow up. Gray lines indicate shared covariance. Only statistically significant paths (P < 0.05) are shown. Model fit indices: CFI: 0.99, RMSA: 0.05, SRMR: 0.025. Data presented are standardized coefficients (standard error). [file 12966_2023_1417_MOESM1_ESM.docx]

Supplementary material.

# Additional tables

# Table S1. Covariance matrices for the daily time-use in movement behaviours.

|  |  | VPA | MPA | LPA | SB | Sleep |
| --- | --- | --- | --- | --- | --- | --- |
| Baseline (4 years old) |  |  |  |  |  |  |
| VPA |  |  | 0.22 | 0.34 | 0.47 | 0.35 |
| MPA |  | 0.22 |  | 0.13 | 0.43 | 0.21 |
| LPA |  | 0.34 | 0.13 |  | 0.21 | 0.09 |
| SB |  | 0.47 | 0.43 | 0.21 |  | 0.19 |
| Sleep |  | 0.35 | 0.21 | 0.09 | 0.19 |  |
|  |  | VPA | MPA | LPA | SB | Sleep |
| Follow up (9 years old) |  |  |  |  |  |  |
| VPA |  |  | 0.17 | 0.35 | 0.44 | 0.37 |
| MPA |  | 0.17 |  | 0.11 | 0.21 | 0.15 |
| LPA |  | 0.35 | 0.11 |  | 0.09 | 0.04 |
| SB |  | 0.44 | 0.21 | 0.09 |  | 0.03 |
| Sleep |  | 0.37 | 0.15 | 0.04 | 0.03 |  |

Note: values close to 0 represent high covariance (dependence) between the variables.

VPA: vigorous physical activity, MPA: moderate physical activity, LPA: light physical activity, SB: sedentary behaviour.

# Table S2. Comparison of baseline demographic characteristics (4 years old) of children included and not included in this study.

|  |  | Included | |  | Not included | | |  |
| --- | --- | --- | --- | --- | --- | --- | --- | --- |
|  |  | n | Value |  | | n | Value | *P* value |
| Age (yrs) |  | 201 | 4.5 (0.1) |  | | 114 | 4.5 (0.1) | 0.950 |
| Height (cm) |  | 201 | 107.4 (4.2) |  | | 114 | 107.9 (4.3) | 0.302 |
| Weight (kg) |  | 201 | 18.2 (2.5) |  | | 114 | 18.5 (2.5) | 0.328 |
| Weight status *n (%)* |  | 201 |  |  | | 114 |  | 0.514 |
| Underweight |  |  | 20 (9.9) |  | |  | 7 (6.1) |  |
| Normal weight |  |  | 166 (82.6) |  | |  | 94 (82.5) |  |
| Overweight |  |  | 13 (6.5) |  | |  | 11 (9.6) |  |
| Obesity |  |  | 2 (1.0) |  | |  | 2 (1.8) |  |
| Maternal education level *n (%)* |  | 201 |  |  | | 114 |  | 0.034 |
| University or higher |  |  | 151 (75.1) |  | |  | 72 (63.2) |  |
| Below university |  |  | 50 (24.9) |  | |  | 42 (36.8) |  |
| *Body composition* |  |  |  |  | |  |  |  |
| FFMI (kg·m^2^) |  | 201 | 11.6 (1.0) |  | | 102 | 11.6 (0.9) | 0.888 |
| FMI (kg·m^2^) |  | 201 | 4.1 (0.9) |  | | 102 | 4.2 (0.9) | 0.383 |
| *Physical fitness* |  |  |  |  | |  |  |  |
| Aerobic (laps) |  | 201 | 5.9 (2.7) |  | | 106 | 5.7 (2.5) | 0.528 |
| Speed-Agility (s) |  | 201 | 18.0 (1.9) |  | | 114 | 18.3 (2.0) | 0.208 |
| Upper-body strength (kg) |  | 201 | 6.4 (1.6) |  | | 112 | 6.5 (1.6) | 0.545 |
| Lower-body strength (cm) |  | 201 | 73.6 (16.0) |  | | 113 | 67.9 (12.9) | 0.001 |

Data presented as mean (SD) unless otherwise stated.


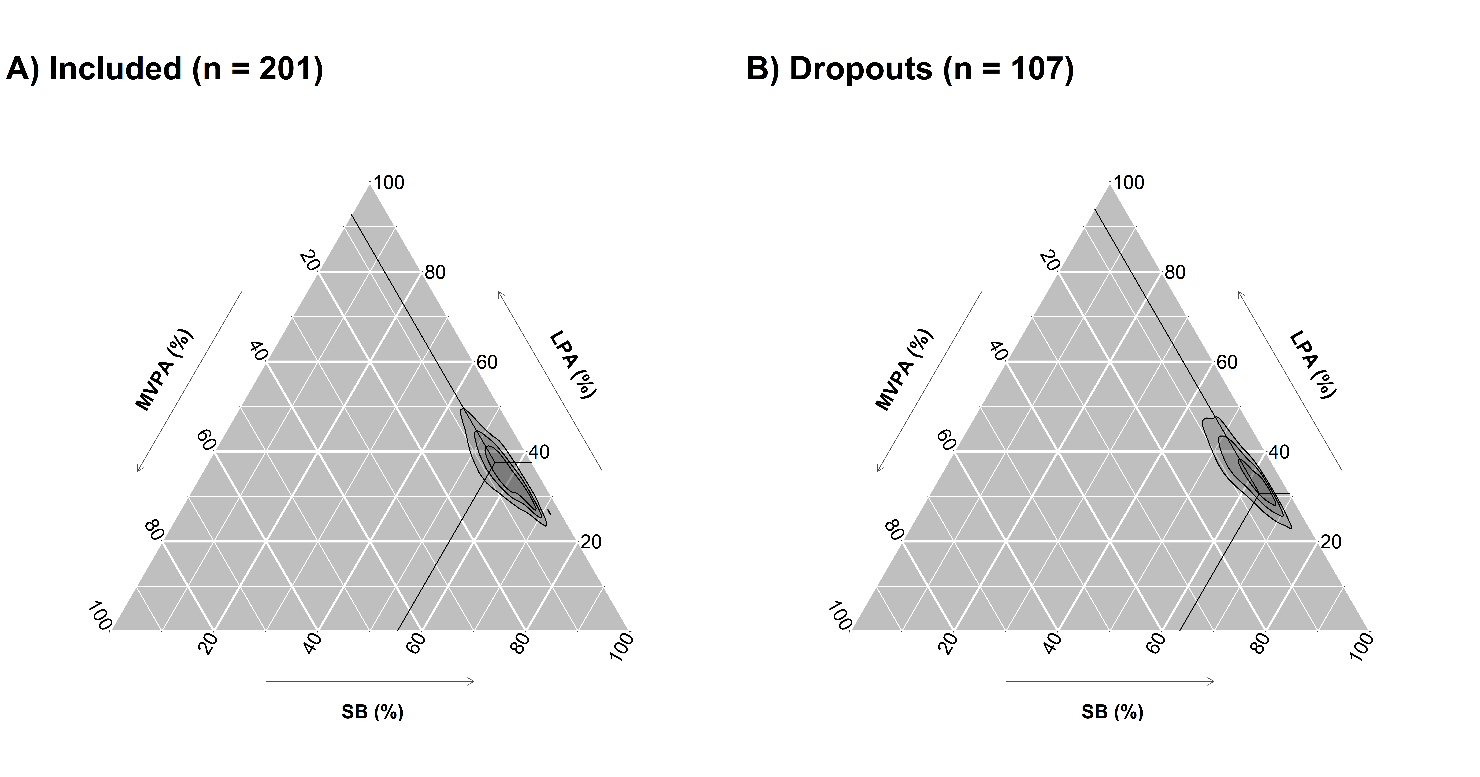


# Figure S1. Ternary plots for the daily time-use in the movement behaviours at 4 years old in the included children (A) and the dropouts (B). The crosshair marks represent the geometric mean of the awake time behaviours at 4 years old in the (A) included children (i.e., VPA: 10 min/day, MPA: 55 min/day, LPA: 348 min/day, SB: 512 min/day, Sleep: 515 min/day) and (B) 9 years old (i.e., VPA: 8.4 min/day, MPA: 51 min/day, LPA: 272 min/day, SB: 562 min/day, Sleep: 645 min/day). Concentric rings represent the 25%, 50% and 75% confidence regions. The difference in the time-use composition of VPA, MPA, LPA, SB, and sleep is not significantly different between the included children and the dropouts (*P* = 0.628).

MVPA: moderate-to-vigorous physical activity, VPA: vigorous physical activity, MPA: Moderate physical activity, LPA: light physical activity, SB: sedentary behaviour.


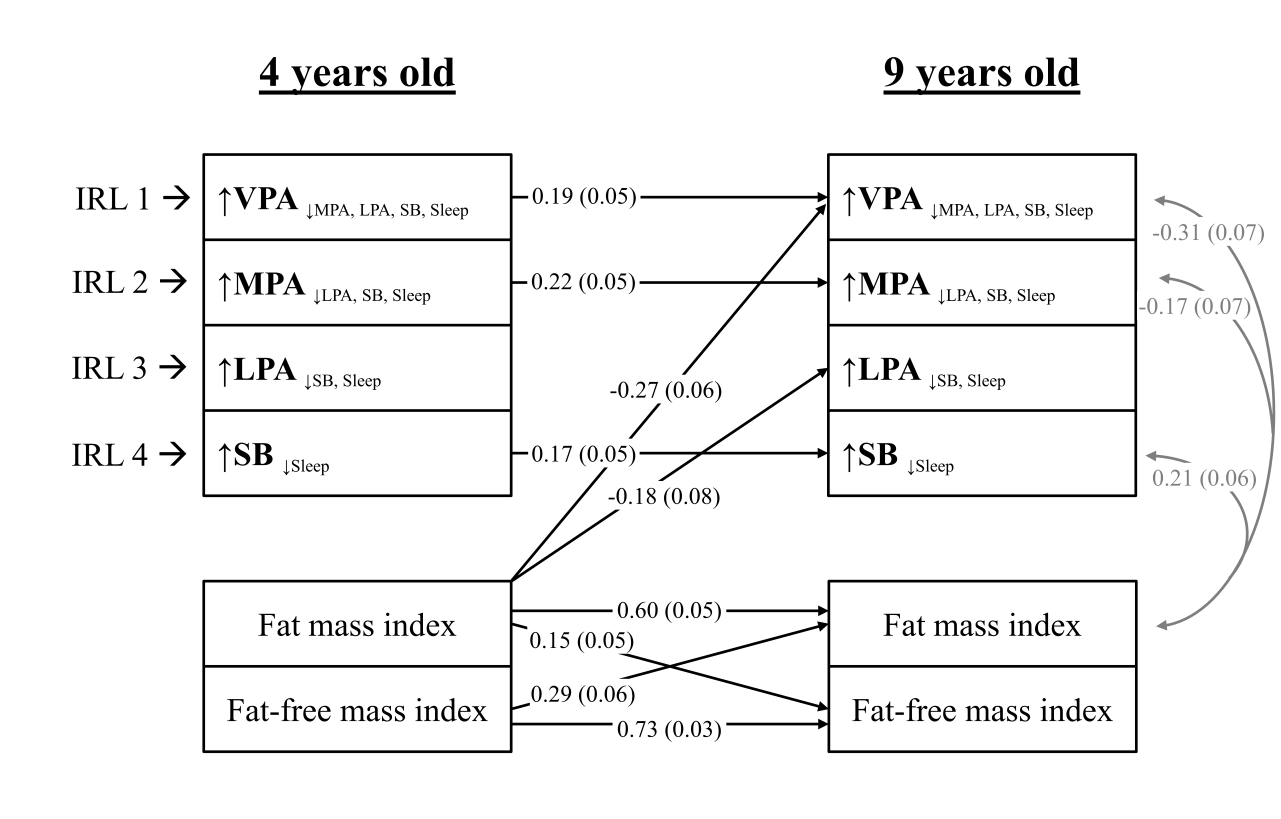


**Figure S2.** Lagged associations of the 24-h movement behaviour composition with body composition and concurrent relationships at 9 years old (n = 196). The model is adjusted for sex and age at baseline and follow up, group allocation, maternal education, and energy intake at baseline and follow up. Gray lines indicate shared covariance. Only statistically significant paths (P < 0.05) are shown. Model fit indices: CFI: 0.99, RMSA: 0.05, SRMR: 0.025. Data presented are standardized coefficients (standard error).


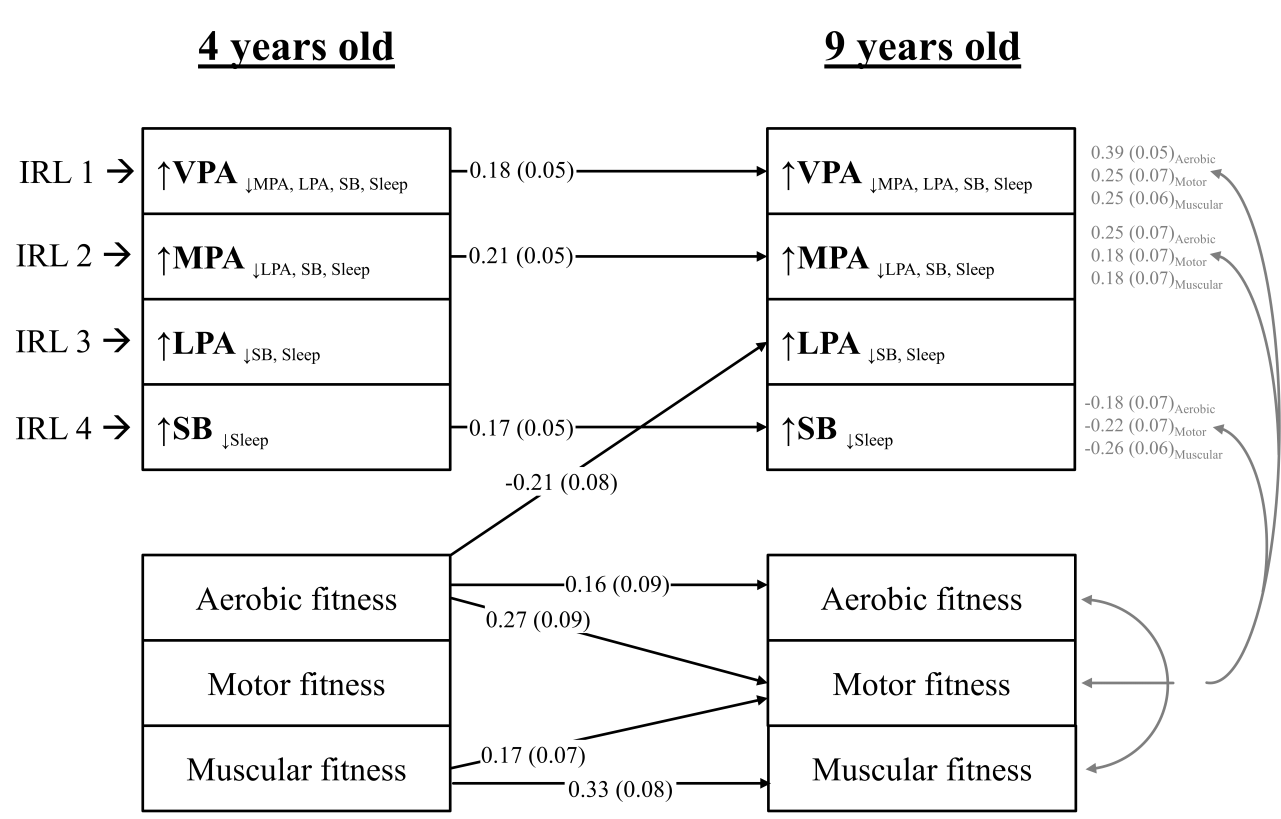


**Figure S3.** Lagged associations of the 24-h movement behaviour composition with physical fitness and concurrent relationships at 9 years old (n = 196). The model is adjusted for sex and age at baseline and follow up, group allocation, maternal education, and energy intake at baseline and follow up. Gray lines indicate shared covariance. Only statistically significant paths (P < 0.05) are shown. Model fit indices: CFI: 0.99, RMSA: 0.05, SRMR: 0.025. Data presented are standardized coefficients (standard error).
